# PARP Inhibitor Olaparib Use in a BRCA1-Positive Patient With Metastatic Triple-Negative Breast Cancer, Without the Initial Use of Platinum-Based Chemotherapy, Showing Significant Rapid Near Resolution of Large Liver Metastasis While Patient Experienced Gout-Like Symptoms

**DOI:** 10.1177/2324709619864989

**Published:** 2019-08-02

**Authors:** Trevanne Matthews Hew, Lara Zuberi

**Affiliations:** 1 University of Florida, Jacksonville, FL, USA

**Keywords:** olaparib, gout, BRCA1, triple-negative breast cancer, PARP inhibitor breast cancer

## Abstract

Triple-negative breast cancer (TNBC) accounts for 20% of breast cancers diagnosed worldwide. This subtype of breast cancer tends to behave more aggressively, and unlike other breast cancer subtypes, there are no standard targeted treatments for most patients. However, up to 20% of patients with TNBC harbor a breast cancer gene *(BRCA)* mutation, particularly in *BRCA1*. For patients who carry this gene mutation, this opens the door for new management options by the use of newer agents such as polyadenosine diphosphate-ribose polymerase (PARP) inhibitors in the metastatic setting. Given that this is uncommon and that PARP inhibitors have only recently received Federal Drug Administration approval, the experience with these drugs is relatively new. In this article, we present a case of a patient treated in this setting with olaparib who developed an unanticipated side effect as a result of the high efficacy of the drug.

## The Case

This is the case of a 62-year-old physician of Polish descent who was diagnosed with bilateral TNBC. Family history was positive for breast cancer in an aunt and a sister. Pathology of the right breast showed infiltrating solid papillary carcinoma with focal necrosis Nottingham grade 3, and for the left breast infiltrating poorly differentiated ductal carcinoma with associated lymphoplasmacytic infiltration, Nottingham grade 3, both TNBCs. Positron emission tomography (PET) scan at diagnosis was negative for distant metastasis.

Soon after she underwent bilateral mastectomy and bilateral sentinel node biopsies. Pathology revealed a right breast 1.3-cm mass, which was estrogen receptor negative, progesterone receptor negative, and human epidermal growth factor receptor 2 (HER-2) negative, that is, triple negative, with negative lymph nodes (N0). The left breast revealed 1.2-cm mass also triple-negative N1 with micro-invasion (mic) without lymphovascular invasion. No metastasis found at that time (M0). Subsequent genetic testing showed that she had BRCA1 mutation. With the diagnosis of bilateral stage 1 breast cancer, right T1c N0 M0, and left T1cN1mic/N0 Mx triple negative, BRCA1 positive, after she completed mastectomy, she was treated with 4 cycles of adjuvant chemotherapy with taxotere and cyclophosphamide. She declined anthracycline chemotherapy.

**Figure 1.**
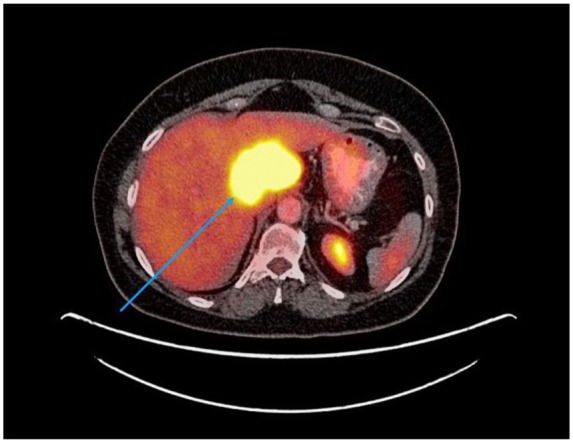
PET scan axial view, 1 year post diagnosis, pre-PARP inhibitor treatment. Blue arrow shows large liver metastasis.

**Figure 2.**
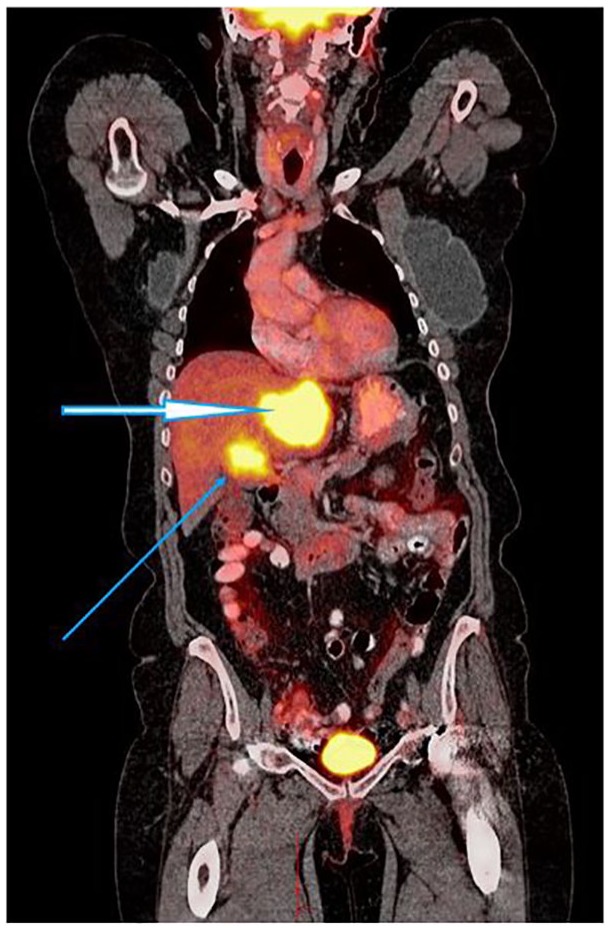
PET scan coronal view, 1 year after diagnosis, prior to PARP inhibitor treatment. Thick and thin arrows showing liver metastasis.

She had regular visits with no issues. However, at one surveillance follow-up visit after 1 year, she complained of mild right upper quadrant pain. This prompted urgent imaging, which revealed large liver metastasis, the largest of which measuring 5 cm. PET scan at this time showed these to be hypermetabolic with no extra hepatic metastasis (see Figures 1 and 2, showing large PET avid liver lesions).

She was offered platinum chemotherapy; however, she declined as she wished to continue working as a physician, which was very important to preserving her quality of life.

Her case was discussed in the tumor board, and consensus was systemic therapy with olaparib based on new evidence of benefit. She was started on olaparib and she initially felt well. However, within days she developed an erythematous rash on her right ankle, which was somewhat painful and tender followed by pain, tenderness, swelling, and erythema in the left big toe, consistent with gout. The patient was asked to stop olaparib for few days and take Benadryl and ibuprofen. Allopurinol was also added.

Uric acid was normal; however, the labs were drawn at the time her symptoms had improved significantly. After the symptoms improved, she was able to restart olaparib, and within a few weeks, the symptoms had completely resolved.

**Figure 3.**
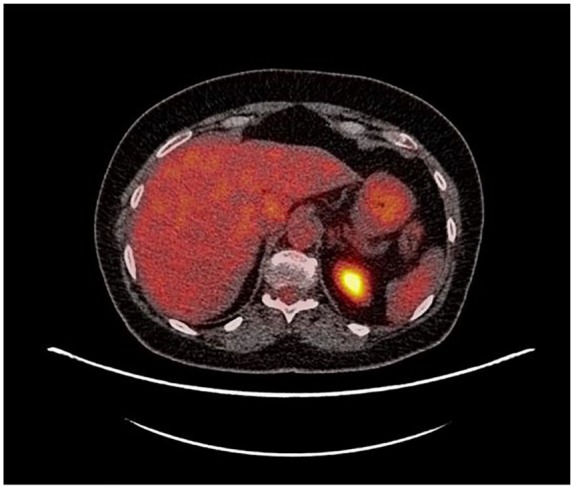
PET scan 3 months post-PARP inhibitor. No metastasis seen in the liver.

**Figure 4.**
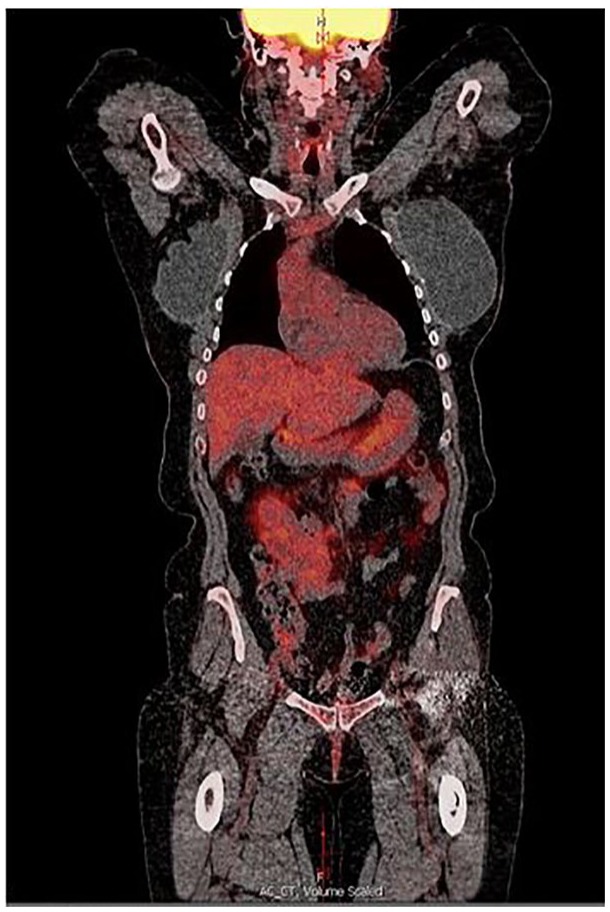
PET scan coronal view, 3 months post-PARP inhibitor treatment. No liver metastasis seen.

Repeat PET scan at 3 months showed near complete response to therapy with significant decrease in the size and near complete resolution of metabolic activity within the 2 large hepatic masses seen prior (see Figures 3 and 4).

## Discussion

For stage IV breast cancer, 5-year overall survival is 27%, and for triple-negative stage IV, it is much less with median survival of 12 to 18 months. TNBC tends to behave more aggressively than other types of breast cancer. Chemotherapy has been the mainstay of systemic treatment, as endocrine and HER-2-directed therapies are ineffective.

Up to 20% of patients with TNBC harbor a breast cancer gene *BRCA* mutation, particularly in *BRCA1*. By contrast, <6% of all breast cancers are associated with a *BRCA* mutation. Most breast cancers that arise in the setting of germline mutation in BRCA1 are triple negative and therefore all patient with TNBC at any age should be offered testing.^1,2^

Based on randomized trials, both platinums and taxanes are considered appropriate first-line therapy. Among those with germline BRCA mutations, the progression-free survival (PFS) was longer with platinum agents than seen with taxanes, making this the class for front-line therapy (OlympiAD Trial). However, with our patient, she refused platinum-based chemotherapy stating that she did not want the side effects affecting her ability to go to work daily as a physician. PARP inhibitor was offered as the alternative management option.

Inhibitors of PARP may be particularly useful in *BRCA-*mutated breast cancer, of which the majority are triple negative. For patients with germline *BRCA* mutations and previously treated HER2-negative, advanced breast cancer (either hormone receptor positive or triple negative), the oral inhibitors of PARP olaparib and talazoparib have shown efficacy.^3^

In the OlympiAD trial, among the subset of 121 *BRCA* mutation carriers with metastatic triple-negative disease, all of whom had received an anthracycline and a taxane in either the adjuvant or metastatic setting, those randomly assigned to olaparib experienced an improved PFS relative to those receiving chemotherapy (hazard ratio for progression or death = 0.43, 95% confidence interval = 0.29-0.63).^4^ The improvements noted with olaparib were stronger in the triple-negative population. Similarly, in the TNBC subgroup of the EMBRACA trial, which also enrolled patients with advanced breast cancer and a germline *BRCA* mutation, talazoparib improved PFS relative to single-agent chemotherapy.^5^ PARP is involved in the molecular events leading to cell recovery from deoxyribonucleic acid (DNA) damage. When *PARP1*, the most abundant member of the PARP family, is inhibited, double-strand DNA breaks accumulate and under normal conditions are repaired via the BRCA pathway-dependent homologous recombination mechanism.^6^ Investigators hypothesized that inhibition of PARP, in combination with DNA-damaging chemotherapeutics, would render tumors lacking *BRCA* function exquisitely sensitive.^7^

Our patient received 4 cycles of Taxotere and cyclophosphamide in the first-line setting for stage 1 disease and faced with metastasis to the liver opted not for platinum chemotherapy but agreed to start on PARP inhibitor. The response to the olaparib she was started on was rapid and dramatic with significant improvement in her abdominal pain and near resolution of the PET avid area noted on PET scan just months later (see Figures 1 to 4).

**Figure 5.**
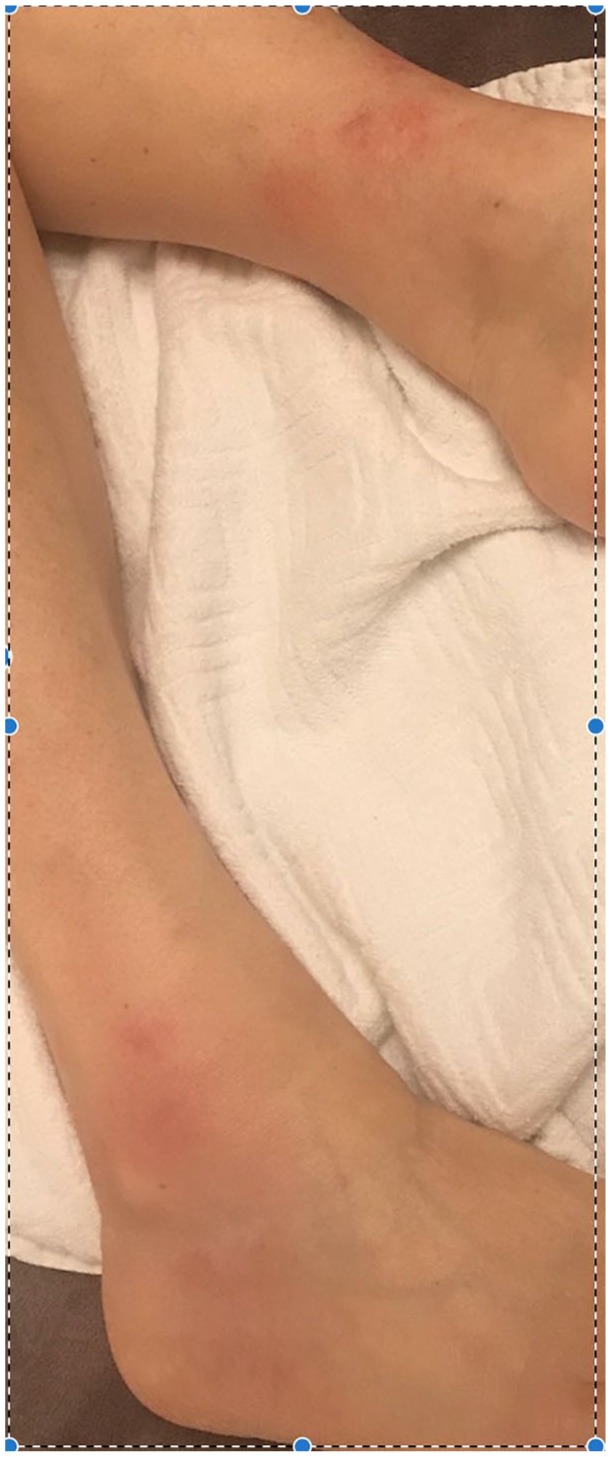
Swelling and erythema to the ankles shown.

Notable during the time of treatment, she experienced what she described as gout-like symptoms. She had sudden onset of right ankle pain and left first metatarsal and metatarsal phalangeal joint pain, which was associated with touch sensitivity erythema and increased warmth (Figure 5). The symptoms only abated when the drug was held for a few days and ibuprofen was taken with allopurinol. Once the medication was restarted, the pain returned but with milder intensity. The pain and symptoms resolved after 3 to 4 weeks.

This gives the thought to the possibility of PARP inhibitor causing such rapid significant tumor destruction in a patient with high tumor burden as seen in our patient to precipitate gout.

Our case highlights the fact that we often do not consider tumor lysis as a consequence of treatment for solid tumors, but rather hematological malignancies. However, with highly efficacious targeted therapy, this is perhaps important to consider, monitor, and manage.

## Conclusion

PARP inhibitors, such as olaparib, have joined a list of new agents accessible for practitioners to use in patients in their fight against cancer. Many have offered new hope for PFS, some in overall survival and in others a cure where there was none prior. With these new drugs, much still is to be known such as immediate and long-term side effects. With time and more experience, we will learn more. This case illustrates the dramatic response to PARP inhibitors and a possible potential side effect to look out for in the future.
